# Characterization of Subtype H6 Avian Influenza A Viruses Isolated From Wild Birds in Poyang Lake, China

**DOI:** 10.3389/fvets.2021.685399

**Published:** 2021-09-13

**Authors:** Zhimin Wan, Qiuqi Kan, Zhehong Zhao, Hongxia Shao, Thomas J. Deliberto, Xiu-Feng Wan, Aijian Qin, Jianqiang Ye

**Affiliations:** ^1^Key Laboratory of Jiangsu Preventive Veterinary Medicine, Key Laboratory for Avian Preventive Medicine, Ministry of Education, College of Veterinary Medicine, Yangzhou University, Yangzhou, China; ^2^Institute of Agricultural Science and Technology Development, Yangzhou University, Yangzhou, China; ^3^Jiangsu Co-innovation Center for Prevention and Control of Important Animal Infectious Diseases and Zoonoses, Yangzhou, China; ^4^Joint International Research Laboratory of Agriculture and Agri-Product Safety, the Ministry of Education of China, Yangzhou University, Yangzhou, China; ^5^Institute of Comparative Medicine, Yangzhou University, Yangzhou, China; ^6^National Wildlife Disease Program, Wildlife Services, Animal and Plant Health Inspection Service, U.S. Department of Agriculture, Fort Collins, CO, United States; ^7^University of Missouri Center for Influenza and Emerging infectious Diseases, University of Missouri, Columbia, MO, United States; ^8^Department of Molecular Microbiology and Immunology, School of Medicine, University of Missouri, Columbia, MO, United States; ^9^Bond Life Sciences Center, University of Missouri, Columbia, MO, United States; ^10^Department of Electrical Engineering and Computer Science, College of Engineering, University of Missouri, Columbia, MO, United States

**Keywords:** H6 subtype, wild birds, Poyang Lake, reassortment, phylogenetic analysis, replication

## Abstract

Subtype H6 avian influenza A viruses (IAVs) are enzootic and genetically diverse in both domestic poultry and wild waterfowl and may cause spillovers in both pigs and humans. Thus, it is important to understand the genetic diversity of H6 IAVs in birds and their zoonotic potential. Compared with that in domestic poultry, the genetic diversity of H6 viruses in wild birds in China has not been well-understood. In this study, five H6 viruses were isolated from wild birds in Poyang Lake, China, and genetic analyses showed that these isolates are clustered into four genotypes associated with reassortments among avian IAVs from domestic poultry and wild birds in China and those from Eurasia and North America and that these viruses exhibited distinct phenotypes in growth kinetics analyses with avian and mammalian cells lines and in mouse challenge experiments. Of interest is that two H6 isolates from the Eurasian teal replicated effectively in the mouse lung without prior adaptation, whereas the other three did not. Our study suggested that there are variations in the mammalian viral replication efficiency phenotypic among genetically diverse H6 IAVs in wild birds and that both intra- and inter-continental movements of IAVs through wild bird migration may facilitate the emergence of novel H6 IAV reassortants with the potential for replicating in mammals, including humans. Continued surveillance to monitor the diversity of H6 IAVs in wild birds is necessary to increase our understanding of the natural history of IAVs.

## Introduction

The influenza A virus (IAV) belongs to the *Orthomyxoviridae* family and contains a genome with eight negative sense, single-stranded RNA segments. Based on the antigenic properties of two surface glycoproteins, hemagglutinin (HA) and neuraminidase (NA), IAVs are classified into 18 HA and 11 NA subtypes (1, 2). Among these subtypes, H1–H16 and N1–N9 have been identified in IAVs recovered from wild birds, especially migratory waterfowl (3). Wild bird migration not only facilitates reassortment among IAVs from different geographic locations but may also introduce these IAVs to domestic animals (e.g., poultry and swine) and humans across regions. Thus, it is important to differentiate typical and atypical influenza occurrences in waterfowl, especially among the interfaces among wild birds, domestic animals, and humans, which provide frontiers for the emergence of potential pandemic IAVs.

Since its first isolation from a turkey in the United States in 1965 (4), subtype H6 avian IAVs have been identified in various migratory waterfowl and domestic poultry in Eurasia and North America (5) and contributed to the genomic exchange and diversity of IAVs in wild birds (2, 6–9). The frequent introductions of H6 IAVs from wild birds to domestic poultry have been documented, but most of these introductions only resulted in limited transmission in domestic poultry. However, H6N2 IAVs were shown to be introduced from Eurasia and subsequently caused outbreaks and became enzootic among domestic poultry in California, USA, from 2000 to 2005 (10, 11). In southern China, in the past two decades, H6 IAV was one of the predominant subtypes in live bird markets (12–16), and some of these H6 viruses recognized human-like glycan receptors (17). In addition, H6 IAVs have been isolated from wild birds in China (16, 18), although the genetic diversity of H6 IAVs in wild birds in China is not well-understood.

Humans can be infected with H6 IAVs through experimental inoculation (19). Previous studies suggested H6 IAVs may replicate well in mice without pre-adaptation, indicating the potential that these viruses can cause cross-species infection in mammals (20, 21). Serological surveillance suggested that veterinarians exposed to H6 IAV-infected domestic birds can be infected with the virus (22). In 2010, after an avian-origin H6N6 swine IAV was isolated from sick pigs in southern China; the seroprevalence ranged from 1.8 to 3.4% in pigs (23, 24). However, ferret experiments showed that this H6N6 virus has limited transmissibility between ferrets either through direct contact or through the inhalation of infectious aerosolized droplets (25). In 2013, an avian-origin H6N1 IAV was reported to cause human infection, but there has been no evidence of subsequent human-to-human transmission (26). Other studies also suggested that humans and other mammals had been exposed to H6 IAVs (27, 28). All these studies suggest that H6 IAVs pose threats to public health and shall be monitored during influenza surveillance at the interface of wild bird and domestic poultry.

In this study, we performed avian influenza surveillance in wild birds at Poyang Lake, the largest freshwater lake in China, and isolated five H6 IAVs. We determined the genetic diversity of these H6 isolates. To understand their zoonotic potential, we characterized the replication ability in both human cells and mice.

## Materials and Methods

### Virus Detection and Isolation

A total of 981 cloacal samples were collected from wild birds in Poyang Lake in spring 2018. RNAs were extracted from these samples, and cDNAs were synthesized by reverse transcription (RT) with the Uni12 primer: 5′-AGCAAAGCAGG-3′ (29), and the M gene was amplified by PCR with the specific primers (30). The positive samples were inoculated into 10-day-old specific-pathogen-free (SPF) embryonated chicken eggs for viral isolation. Allantoic fluid was harvested after 72 h of culture and tested by hemagglutination assay with 0.5% chicken red blood cells.

### Genomic Sequencing, Phylogenetic, and Genotype Analyses

Viral RNAs were extracted from HA-positive allantoic fluid samples, and cDNAs were synthesized as mentioned above. PCR was performed for each of the eight genes by using gene segment-specific primers as described elsewhere (30), and the PCR products were sequenced by Sanger sequencing.

To identify potential precursor viruses, we used BLAST to search genetically similar genes across GenBank. All these genes that we identified and their associated genomic sequences were downloaded and included in the phylogenetic analyses. In addition, the genomic sequences for additional IAVs, particularly those causing swine and human cases in China, were obtained from the Global Initiative on Sharing Avian Influenza Data (GISAID).

Multiple sequence alignments were carried out by using the FASTA module embedded in BioEdit version 7.2.1. Phylogenetic trees were constructed by using the neighbor-joining method, with bootstrap analysis (1,000 replicates), in MEGA-X (31). To perform time to most recent common ancestor analyses, a maximum likelihood tree derived from the IQ tree was used for Treetime (0.8.1) analysis. When the results show that the sampling dates could be utilized for Beast analysis, BEAST (v1.10.4) was executed with the Bayesian skygrid coalescent model under the uncorrelated relaxed clock using Markov chain Monte Carlo (MCMC). The MCMC chain length was set as 200 million generations and sampled every 20,000 steps. Logfile was inspected by the tracer (v1.7.1) to guarantee all the effective sample sizes >200 (ESS >200). Lastly, the MCC tree was obtained from TreeAnnotator (v1.10.4) and visualized in Figtree (v1.4.4).

The genetic clusters were assigned based on the topology of the phylogenetic trees with a minimal bootstrap value of 70, and the genes within the same genetic cluster shall have a minimal nucleotide sequence identity of 95% as described (18, 32). The genotypes were determined by unique combinations of genetic clusters across eight genetic segments.

### 50% Egg Infection Dose

Groups of 10-day-old SPF embryonated chicken eggs were infected with serial 10-fold dilutions of H6 IAVs and incubated at 37°C for 72 h. The allantoic fluid was harvested and tested by hemagglutination assay. The 50% egg infection dose (EID_50_) value was calculated using the method of Reed and Muench (33).

### 50% Tissue Culture Infection Dose

Moreover, 90% confluent DF1, MDCK, or A549 cells were infected with serial 10-fold dilutions of viruses and incubated at 37°C. After 2 h, the virus inoculum was removed, and the cells were washed twice with phosphate buffer solution, maintained in opti-MEM media containing 1 μg/ml TPCK-trypsin, and incubated at 37°C for 72 h. The supernatant was tested by hemagglutination assay. The 50% tissue culture infection dose (TCID_50_) value was calculated using the method of Reed and Muench (33).

### *Vitro* Growth Kinetics

A total of 90% confluent DF-1, MDCK, or A549 cells were infected with each H6 IAV isolate at a multiplicity of infection of 0.01 and maintained in opti-MEM media containing 1 μg/ml TPCK-trypsin and incubated at 37°C. The cell culture supernatants were harvested at 6, 12, 24, 48, and 72 h post-infection (hpi), and then virus titers were determined by TCID_50_ in DF-1, MDCK, or A549 cells. The experiments were performed in triplicates.

### Animal Study

Five-week-old male Balb/c mice (Yangzhou University) were randomly distributed into six groups (12 mice/group). The mice were anesthetized with isoflurane and each infected with H6 IAV at 10^5^ EID_50_ in a volume of 25 μl by intranasal inoculation. At 3 and 5 days post-infection (dpi), three mice from each group were sacrificed by receiving an overdose of isoflurane and then humanely euthanized, and the lungs were collected to determine virus titers in MDCK cells. The remaining six mice in each group were monitored daily for body weight loss and clinical signs. The mice with body weight loss of more than 25% will be anesthetized and euthanized.

### Ethics Statement

The animal study was performed in accordance with the institutional animal guidelines, and protocol #06R015 was approved by the Animal Care Committee at Yangzhou University, China. The ethical permission code (no. SYXK-SU-2017-0007, licensed on July 19, 2022) was provided by the Animal Ethics Committee of Yangzhou University.

## Results

### Influenza Virus Isolation and Subtype Identification

A total of 35 IAV-positive samples were identified from 981 cloacal samples collected from wild birds in Poyang Lake. From these samples, seven IAV isolates were obtained, including one H12N1, one H9N2, and five H6 viruses. We sequenced the full genomes of the five H6 isolates: A/Eurasian teal/Jiangxi/2018WB0049/2018 (H6N2) (E-Teal/49, H6N2), A/Eurasian wigeon/Jiangxi/2018WB0158/2018 (H6N2) (E-Wigeon/158, H6N2), A/Eurasian wigeon/Jiangxi/2018WB0266/2018 (H6N2) (E-Wigeon/266, H6N2), A/Eurasian teal/Jiangxi/2018WB0417/2018 (H6N2) (E-Teal/417, H6N2), and A/Greater White-fronted goose/Jiangxi/2018WB0740/2018 (H6N1) (GWF-Goose/740, H6N2). All the sequences were submitted to the GISAID (accession no.: EPI1868495-EPI1968523).

### Phylogenetic Analyses of H6 IAVs

**Figure 1:**
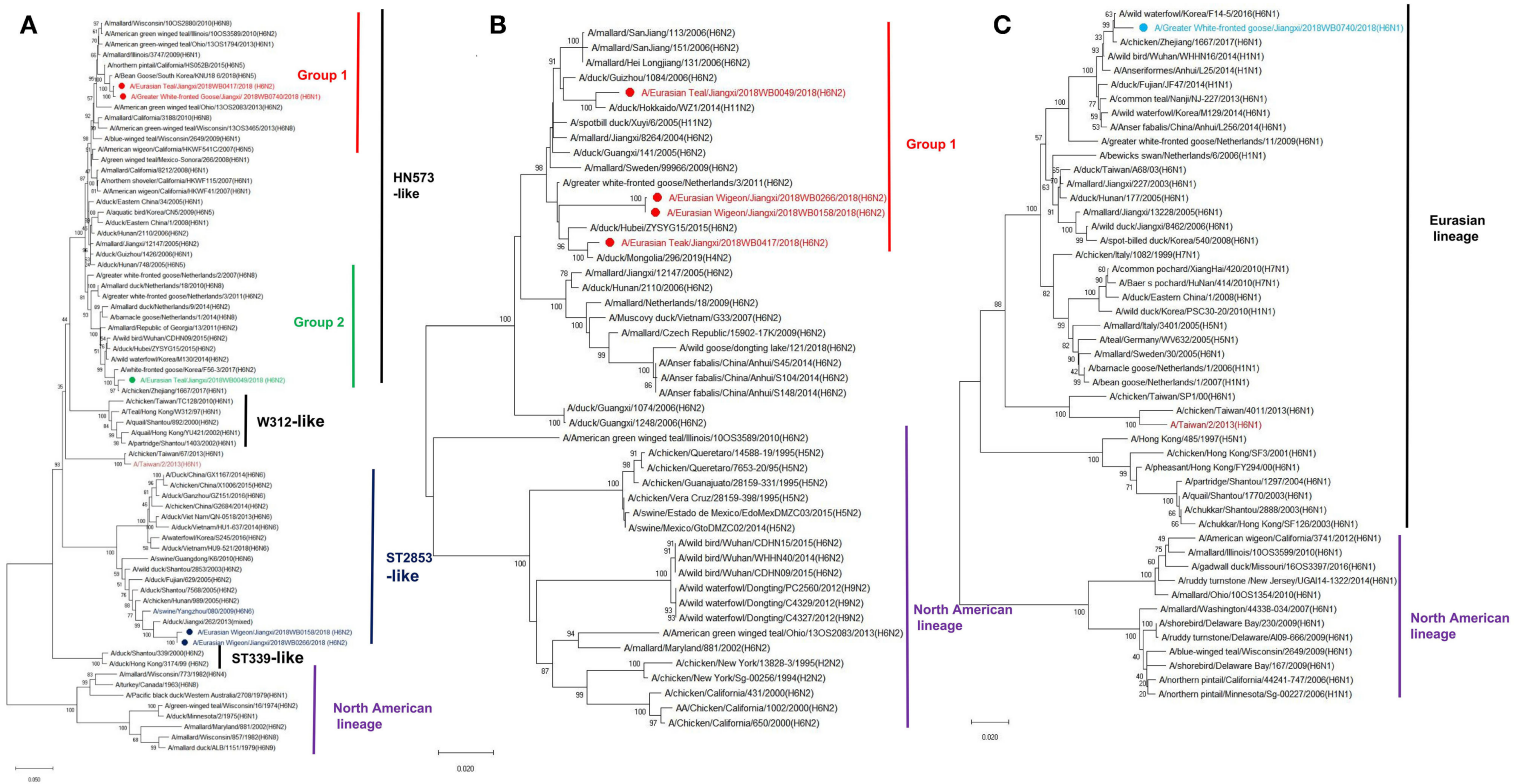
Phylogenetic trees of surface protein genes H6 **(A)**, N1 **(B)**, and N2 **(C)** of H6 avian influenza A viruses (IAVs) isolated from wild birds, Poyang Lake, China. The trees were generated by the maximum likelihood method with the MAGE X software. Bootstrap supports were computed with 1,000 replicates. The scale bar shows the average number of substitutions per nucleotide site. The viruses from this study were labeled in red, green, wathet blue, and blue; the H6 IAV that caused a human infection in Taiwan was marked in mauve, and two H6 viruses that caused spillover cases to domestic swine in southern China were highlighted in blue.

Phylogenetic analyses demonstrated that there was a substantial genetic diversity among these five H6 isolates. The HA genes from E-Teal/417 and GWF-Goose/740 were genetically associated with a bean goose isolate [A/Bean Goose/South Korea/KNU18 6/2018 (H6N5)] and H6 IAVs circulated in the wild bird population from North America, which were initially introduced from Eurasia to North America perhaps in the late 1990s through intercontinental bird migration and then caused outbreaks in domestic poultry in California from 2000 to 2005 (Figure 1A and Supplementary Figure S1) (10, 11, 34). In contrast, the HA genes of E-Wigeon/158 and E-Wigeon/266 were genetically close to H6 viruses circulating in domestic poultry in Southeast China and two avian-origin H6N6 swine IAVs (24, 35), whereas that of E-Teal/49 was genetically close to the HA of H6 viruses circulating in the wild bird population across Eurasia (Figure 1A) and share the same ancestor with A/chicken/Zhejiang/1667/2017 (H6N1). The potential progenitor genes for these HA were shown in Supplementary Table S1 with a nucleotide identity ranging from 99.03 to 99.46%. Of note is that the HA genes of these H6 isolates were not genetically associated with A/Taiwan/2/2013(H6N1), a human isolate report in 2013 (26).

**Figure 2:**
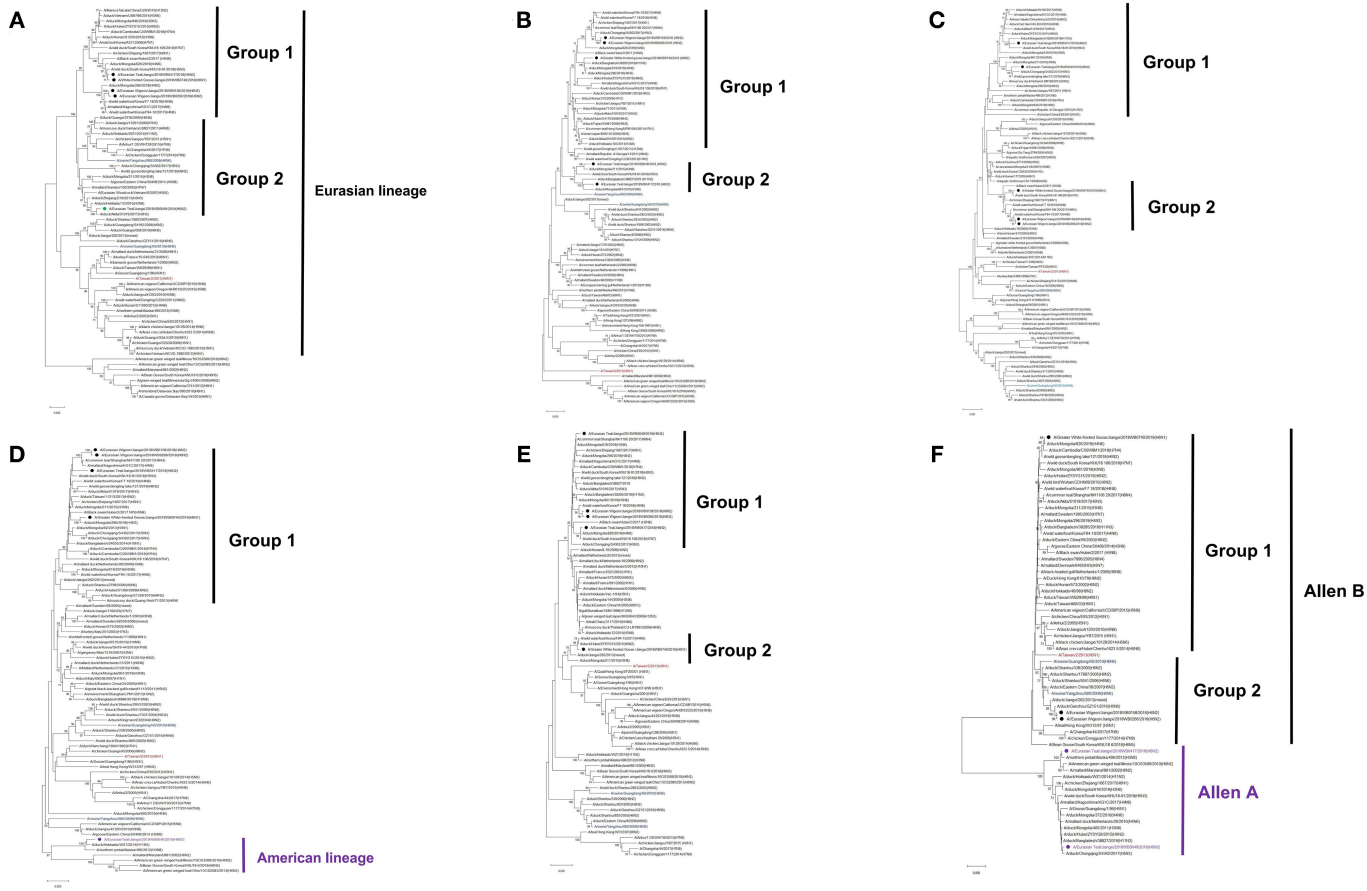
Phylogenetic trees of internal genes PB2 **(A)**, PB1 **(B)**, PA **(C)**, NP **(D)**, MP **(E)**, and NS **(F)** of H6 influenza A virus (IAV) isolates in Poyang Lake, China. The trees were generated by the maximum likelihood method with the MAGE X software. The viruses from this study were labeled in red, green, purple; the H6 IAV that caused a human infection in Taiwan was marked in mauve, and two H6 viruses that caused spillover cases to domestic swine in southern China were highlighted in blue.

Both N2 and N1 of these five H6 isolates were genetically associated with the NA genes of H6N2 and H6N1 IAVs circulating in domestic poultry and wild bird population in Eurasia (Figures 1B,C). Similarly, the majority of the internal genes of these isolates were genetically close to those of viruses circulating in Eurasia, including those from the genetic clades containing H5Nx and H9N2 viruses. Of interest is that NP and NS of E-Teal/49 and NS of E-Teal/417 were genetically close to a group of avian IAVs from East Asia and North America (Figure 2).

Based on the tree topology of each gene segment and on their nucleotide sequence identities, each gene segment of these five isolates was separated into two or three groups, and a total of four genotypes were identified. E-Wigeon/158 and E-Wigeon/266 belong to the same genotype, but the other three fall in three distinct genotypes (Table 1).

**Table 1:**
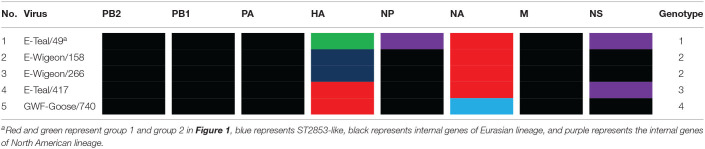
The genotypes of H6 avian influenza A viruses isolated from wild birds, Poyang Lake.

### Molecular Characterization of the Viral Genes of H6 IAVs

Molecular characterization revealed that the receptor binding sites of the five isolates were conserved, with typical wild bird-origin residues (Table 2) that predict the preferential binding to α2,3-linked sialic acid receptors (avian-like glycan receptors) (36); none of these five isolates bear any of the previously reported mutations that can increase the binding affinity of H6 IAV to α2,6-linked sialic acid receptors (human-like glycan receptors, e.g., P186L, E190V, G225D, Q226L, and G228S) (Table 3). Further analyses suggested these wild bird-origin H6 isolates have distinct patterns for the reported disease markers: although E627K and D701N in PB2 for pathogenesis and H274Y and R292K in NA and S31N in the M2 for drug resistance were not found, other mutations for pathogenesis, i.e., A515T in PA, N30D and T215A in M1, and I106M in NS1, were found in all the five H6 isolates. Another genetic marker for pathogenesis, P42S in NS1, was found in E-Wigeon/158, E-Wigeon/266, and GWF-Goose/740 but not in the two Eurasia teal isolates (Table 3). Taking these together, molecular characterization suggested that these five H6 isolates were less pathogenic and highly sensitive to antivirals but with potential diversity in pathogenesis in mammals.

**Table 2:** Amino acids at the cleavage and receptor-binding sites in hemagglutinin (HA) from H6 avian influenza A viruses.

**Virus strain**	**HA cleavage**	**The conserved residues involving receptor binding**							**The edge of receptor-binding site**
H3 number	320–329	98	153	155	183	190	194	195	220–229
H6 number	339–344	107	166	168	197	204	208	209	234–243
E-Teal/49	PQI**G**TR	Y	W	I	H	E	L	Y	RPAVNGQRGR
E-wigeon/158	PQIETR	Y	W	**V**	H	E	L	Y	RPAVNGQRGR
E-Wigeon/266	PQIETR	Y	W	**V**	H	E	L	Y	RPAVNGQRGR
E-Teal.417	PQIETR	Y	W	I	H	E	L	Y	RPAVNGQRGR
GWF-Goose/740	PQIETR	Y	W	I	H	E	L	Y	RPAVNGQRGR

*The residue in bold indicates differences from other strains*.

**Table 3:** Molecular characterization of H6 avian IAVs.

**Protein**	**Genetic marker**	**Virus**					**Function**
		**E-Teal/49**	**E-Wigeon/158**	**E-Wigeon/266**	**E-Teal/417**	**GWF-Goose/740**	
PB2	E627K	E	E	E	E	E	Mammalian adaption mutations (37, 38)
	D701N	D	D	D	D	D	
PA	A515T	T	T	T	T	T	Increased polymerase activity in mammalian cells (39)
HA	P186L^a^	P	P	P	P	P	Increased H6N1 binding to mammalian receptor (40)
	E190V	E	E	E	E	E	Increased H6N2 binding to mammalian receptor (41)
	G225D	G	G	G	G	G	Increased H6N1 virus binding to mammalian receptor (42)
	Q226L	Q	Q	Q	Q	Q	Increased H6N2 virus binding to mammalian receptor (41)
	G228S	G	G	G	G	G	Decreased H6N2 virus binding to α 2.3 (41)
NA	H274Y^b^	H	H	H	H	H	Reduced H6N2 susceptibility to oseltamivir (43, 44)
	R292K	R	R	R	R	R	
M1	N30D	D	D	D	D	D	Increased pathogenesis in mice (45)
	T215A	A	A	A	A	A	
M2	S31N	S	S	S	S	S	Resistance to amantadine and remantadine (46)
NS1	P42S	**A**	**S**	**S**	**A**	**S**	Increased pathogenesis in mice (47, 48)
	I106M	**M**	**M**	**M**	**M**	**M**	

*The residues in bold indicate mutant residues. ^a^H3 number.*

*^b^N2 number.*

### H6 IAV Replication Kinetics in DF-1, MDCK, and A549 Cells

**Figure 3:**
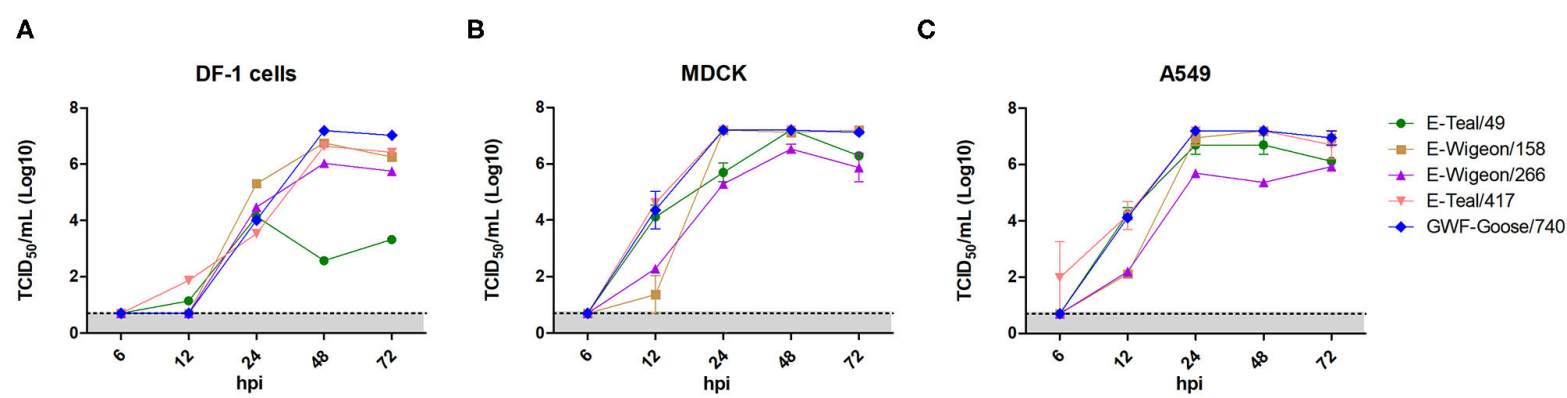
Growth kinetics of H6 avian influenza from wild birds, Poyang Lake. Viral growth kinetics was determined for each virus in DF-1 **(A)**, MDCK **(B)** and A549 **(C)** after inoculation at an MOI of 0.01. Supernatant samples were collected at 6, 12, 24, 48 and 72 hpi, and viral titers were measured in DF-1, MDCK or A549 cells.

To identify the phenotypic diversity of these H6 IAVs in avian and mammalian cells, we examined their growth kinetics in DF-1, MDCK, and A549 cells. The results showed that E-Teal/49 grew well in MDCK and A549 cells, but not in DF-1 cells, and that the other three isolates replicated efficiently in all three cell lines (Figure 3). In DF-1 cells, the three isolates (E-Wigeon/155, E-Wigeon/266, and GWF-Goose/740) peaked at 48 hpi with titers of about 10^7^ TCID_50_/ml (E-Wigenon/155 and GWF-Goose/740) and 10^6^ TCID_50_/ml (E-Wigenon/266) (Figure 3A). In MDCK and A549cells, four (E-Teal/49, E-Wigeon/158, E-Teal/417, and GWF-Goose/740) peaked at 48 h post-inoculation with a titer of >10^7^ TCID_50_/ml, whereas the peak titers of E-Wigeon/266 at 48 hpi were ~10^6^ TCID_50_/ml at 48 hpi in MDCK cells and at 72 hpi in A549 cells (Figures 3B,C). These results showed that all H6 IAVs can replicate efficiently in two tested mammalian cell lines. Of interest is that E-Teal/49 replicates efficiently only in two tested mammalian cell lines but not in avian DF-1 cells.

### Two H6 IAVs Replicated Effectively in Mice Without Prior Adaptation

**Figure 4:**
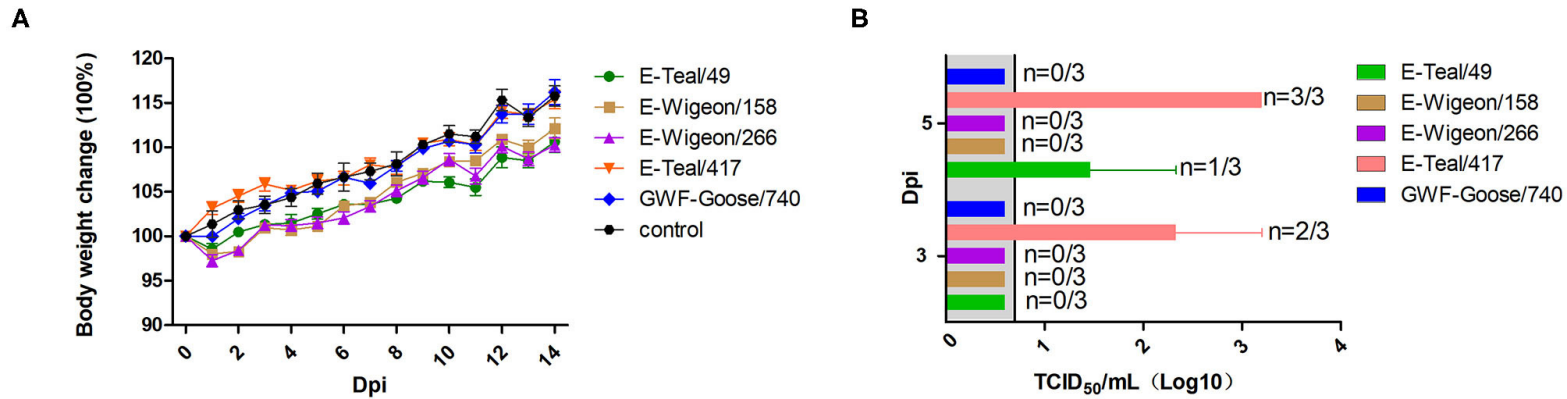
Body weight change and viral replication in mice infected with H6 influenza A viruses (IAVs) from wild birds, Poyang Lake. Five-week-old Balb/c mice were infected with 10^5^ 50% egg infection dose of each virus. Body weight **(A)** and virus replication **(B)** in the lung were determined. The body weight **(A)** of mice was observed over 14 dpi. Lungs **(B)** were collected at 3 and 5 dpi, and viral titers were measured in MDCK cells.

To further investigate the infection potential of these H6 isolates to infect in mammals, 5-week-old Balb/c mice were inoculated intranasally with 10^5^ EID_50_ of each H6 isolate. As shown in Figure 4A, the infected mice showed neither weight loss (Figure 4A) nor any clinical signs of disease. Notably, viral titration showed that E-Teal/49 was detected in the lungs of the infected mice on 3 dpi, and E-Teal/417 could be detected on 3 and 5 dpi; the virus titers were approximately 10^3^ TCID_50_/ml (Figure 4B), whereas the other three H6 IAVs could not be detected in the lungs of the infected animals. These results showed that two H6 isolates from the Eurasian teal were able to replicate in mice without any prior adaptation.

## Discussion

As the natural host for IAVs, wild aquatic birds act as a reservoir for the transmission of IAV genes between species (49, 50). Poyang Lake is on the East Asian-Australasian Flyway of migratory birds, which provides important stopover for birds migrating every year. It plays a crucial role in viral gene reassortment of IAVs. In this study, we isolated five novel H6 IAVs from wild birds in Poyang Lake, which were grouped into four genotypes by phylogenetic analyses (Table 1). In recent years, several reports have shown that H6 reassorts were detected in wild birds, with gene segments from both wild birds and domestic poultry, and these reassortment events not only occurred across Eurasia but also between Eurasia and North America (e.g., through the East Asian-Australasian Flyway) (16, 18, 51, 52). Our data from this study further documented that these five H6 IAVs originated from a complicated reassortment between poultry and wild birds from China and other regions across the Eurasian and North American continents (Figures 1, 2 and Supplementary Table S1).

In the animal study, the two H6 IAVs isolated from teals replicated effectively in mice without prior adaptation. We compared the protein sequences of the teal-origin H6 IAVs with the other three viruses. PB2, PB1, and NP have one common amino acid mutation, respectively (PB2 I451V, PB1 D172E, and NP K458R). There are two common amino acid mutations in PA (E101D and V387) and five mutations in N2 (N127D, I265V, N358S, I380V, and N401S). Interestingly, the NS genes of the two teal-origin H6 IAVs were genetically associated with those reported in East Asia and North America, and those from the other three H6 isolates with those reported from across Eurasia (Figure 2), which have very low amino acid homology. However, the reason that caused the two teal-origin H6 IAV to be replicated effectively in mice needs to be further elucidated.

Of interest is that the five H6 isolates had distinct replication abilities in both different cells and mice, especially E-Teal/49, which did not grow well in DF-1 cells. Molecular analyses suggested that the receptor binding sites were conserved across these H6 isolates (Table 3). Thus, such distinct growth phenotypes were more likely to be associated with the genetic constellation, especially the RNP complex, but not with the receptor binding properties (Figure 2). This study is consistent with a recent study which reported on the wild bird-origin H4N6 viruses that had a high diversity in replication phenotypes in epithelial cells of the swine upper respiratory tract (53). Genetic analyses revealed that the genetic constellation of the RNP complex, rather than the receptor binding properties, was a major factor contributing to the observed phenotypic diversity (53). Nevertheless, the molecular basis for the efficient viral replication in DF-1 and A549 cells and the mouse lung of these H6 IAVs without prior adaptation needs to be further elucidated.

In summary, this study suggested that phenotypic variants exist among genetically diverse H6 IAVs in wild birds and that both intra- and inter-continental movements of IAVs through wild bird migration facilitate the emergence of genetically and phenotypically diverse H6 IAVs. Continued surveillance of the diversity of H6 IAVs in wild birds, especially those unrepresentative regions such as China, is necessary to enhance our understanding of the natural history of IAVs.

## Data Availability Statement

The raw data supporting the conclusions of this article will be made available by the authors, without undue reservation.

## Ethics Statement

The animal study was reviewed and approved by Animal Ethics Committee of Yangzhou University in China.

## Author Contributions

X-FW, AQ, and JY conceived and designed the experiments. ZW, QK, ZZ, and HS performed the experiments. ZW analyzed the data. ZW, X-FW, and TD contributed to the writing of the manuscript. All the authors read and approved the final manuscript.

## Conflict of Interest

The authors declare that the research was conducted in the absence of any commercial or financial relationships that could be construed as a potential conflict of interest.

## Publisher's Note

All claims expressed in this article are solely those of the authors and do not necessarily represent those of their affiliated organizations, or those of the publisher, the editors and the reviewers. Any product that may be evaluated in this article, or claim that may be made by its manufacturer, is not guaranteed or endorsed by the publisher.
