# Supplementary material for: Characterization of Subtype H6 Avian Influenza A Viruses Isolated From Wild Birds in Poyang Lake, China
Source: Front Vet Sci. 2021 Sep 13;8:685399. doi: 10.3389/fvets.2021.685399 (PMC8473872; doi:10.3389/fvets.2021.685399)
Supplement: Supplementary file 2 [file Table_1.DOCX]

Supplementary Material

**Table S1.** Nucleotide identities of the highest homologs in GISAID database with the 5 H6 viruses.

| Gene | Virus with the highest nucleotide identity | Homology | Virus with the highest nucleotide identity | Homology | | Virus with the highest nucleotide identity | Homology | Virus with the highest nucleotide identity | Homology |
| --- | --- | --- | --- | --- | --- | --- | --- | --- | --- |
|  |  | E-Teal/49 |  | E-Wigeon/158 | E-Wigeon/266 |  | E-Teal/417 |  | GWF-Goose/740 |
| PB2 | A/spotbill_duck/Korea/WA159/2018 (A/H5N6) | 99.24% | A/duck/Bangladesh/  2019 (A/H2N2) | 99.3% | 99.04% | A/mallard/Anhui/1-451/2019 (A/H6N2) | 99.38% | A/mallard/Anhui/1-451/2019 (A/H6N2) | 99.38% |
| PB1 | A/Eurasian wigeon/Shanghai  /NH101834 /2017 (H11N2) | 99.65% | A/duck/Chongqing/S4362/  2017 (A/H5N3) | 98.86% | 98.94% | A/mallard/Anhui/3-617/2019 (A/H6N1) | 98.42% | A/spot-billed duck/ Korea/ A45-1/2017 (A/H5N2) | 98.77% |
| PA | A/duck/Guangdong/H31/2020 (A/H3N8) | 99.35% | A/wildwaterfowl/Korea/  F94-10/2017 (A/H4N6) | 99.86% | 99.81% | A/duck/Bangladesh/38827/2019(A/H11N3) | 99.54% | A/environment/Kagoshima/KU-4a/2018 (A/H6N2) | 99.44% |
| HA | A/chicken/Zhejiang/1667/  2017(A/H6N1) | 99.03% | A/duck/Guangdong/11.18_SZBJ002-O/2016 (A/H6N6) | 96.60% | 96.70% | A/northern pintail/Alaska/U  GAI17-4733/2017 (A/H6N5) | 99.46% | A/northern pintail/Alaska/UGA I17-4733/2017(A/H6N5) | 99.33% |
| NP | A/common teal/Shanghai/  NH101807/2017(A/H12N2) | 99.59% | A/common teal/Shanghai  /NH1106-20/2017(A/H8N4) | 98.74% | 98.61% | A/duck/Bangladesh/38285/ 2019(A/H11N3) | 99.07% | A/duck/Mongolia/296/2019(A/H4N2) | 99.14% |
| NA | A/duck/Hokkaido/W105/  2017(A/H5N2) | 99.43% | A/wild bird/ China/Y13/  2019(A/H4N2) | 98.90% | 98.85% | A/duck/Mongolia/296/  2019(A/H4N2) | 99.43% | A/environment/Kagoshima/KU-J2/2018 (A/H4N1) | 99.20% |
| M | A/duck/Mongolia/619/  2019(A/H3N6) | 99.67% | A/wild waterfowl / Korea/F7-18/2018(A/H4N8) | 99.46% | 99.36% | A/duck/Mongolia/826/  2019(A/H4N6) | 99.79% | A/mallard/Korea/F94-16/  2017(A/H4N6) | 99.67% |
| NS | A/spot-billed duck/Korea/H10-1/2017 (A/H5N3) | 99.51% | A/duck/Zhejiang/09.14_HZBX011-O/2018 (A/H0) | 99.39% | 99.39% | A/duck/Gunma/1/2016 (A/H3N8) | 99.51% | A/Bean Goose/South Korea  /KNU18-86/2018(A/H5N2) | 99.88% |

**Figure S1.** The TMRCA-BEAST analysis of HA genes H6 avian IAVs isolated from wild birds, Poyang Lake, China.
