# Supplementary material for: Characterization of Subtype H6 Avian Influenza A Viruses Isolated From Wild Birds in Poyang Lake, China
Source: Front Vet Sci. 2021 Sep 13;8:685399. doi: 10.3389/fvets.2021.685399 (PMC8473872; doi:10.3389/fvets.2021.685399)
Supplement: Supplementary file 1 [file Data_Sheet_1.PDF]

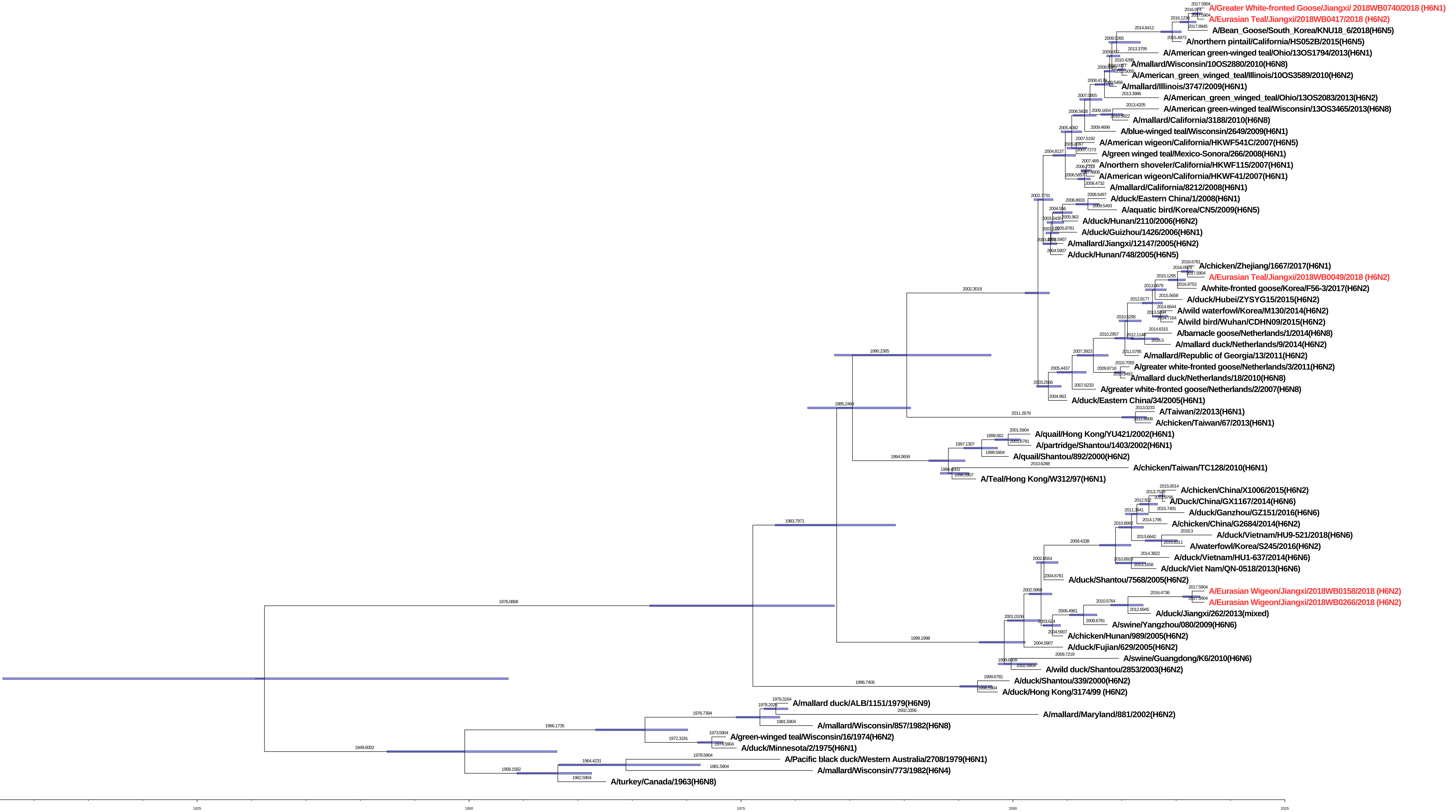
A/Greater White-fronted Goose/Jiangxi/ 2018WB0740/2018 (H6N1)
A/Eurasian Teal/Jiangxi/2018WB0417/2018 (H6N2)
A/Bean_Goose/South_Korea/KNU18_6/2018(H6N5)
A/northern pintail/California/HS052B/2015(H6N5)
A/American green-winged teal/Ohio/13OS1794/2013(H6N1)
A/mallard/Wisconsin/10OS2880/2010(H6N8)
A/American_green_winged_teal/Illinois/10OS3589/2010(H6N2)
A/mallard/Illinois/3747/2009(H6N1)
A/American_green_winged_teal/Ohio/13OS2083/2013(H6N2)
A/American green-winged teal/Wisconsin/13OS3465/2013(H6N8)
A/mallard/California/3188/2010(H6N8)
A/blue-winged teal/Wisconsin/2649/2009(H6N1)
A/American wigeon/California/HKWF541C/2007(H6N5)
A/green winged teal/Mexico-Sonora/266/2008(H6N1)
A/northern shoveler/California/HKWF115/2007(H6N1)
A/American wigeon/California/HKWF41/2007(H6N1)
A/mallard/California/8212/2008(H6N1)
A/duck/Eastern China/1/2008(H6N1)
A/aquatic bird/Korea/CN5/2009(H6N5)
A/duck/Hunan/2110/2006(H6N2)
A/duck/Guizhou/1426/2006(H6N1)
A/mallard/Jiangxi/12147/2005(H6N2)
A/duck/Hunan/748/2005(H6N5)
A/chicken/Zhejiang/1667/2017(H6N1)
A/Eurasian Teal/Jiangxi/2018WB0049/2018 (H6N2)
A/white-fronted goose/Korea/F56-3/2017(H6N2)
A/duck/Hubei/ZYSYG15/2015(H6N2)
A/wild waterfowl/Korea/M130/2014(H6N2)
A/wild bird/Wuhan/CDHN09/2015(H6N2)
A/barnacle goose/Netherlands/1/2014(H6N8)
A/mallard duck/Netherlands/9/2014(H6N2)
A/mallard/Republic of Georgia/13/2011(H6N2)
A/greater white-fronted goose/Netherlands/3/2011(H6N2)
A/mallard duck/Netherlands/18/2010(H6N8)
A/greater white-fronted goose/Netherlands/2/2007(H6N8)
A/duck/Eastern China/34/2005(H6N1)
A/Taiwan/2/2013(H6N1)
A/chicken/Taiwan/67/2013(H6N1)
A/quail/Hong Kong/YU421/2002(H6N1)
A/partridge/Shantou/1403/2002(H6N1)
A/quail/Shantou/892/2000(H6N2)
A/chicken/Taiwan/TC128/2010(H6N1)
A/Teal/Hong Kong/W312/97(H6N1)
A/chicken/China/X1006/2015(H6N2)
A/Duck/China/GX1167/2014(H6N6)
A/duck/Ganzhou/GZ151/2016(H6N6)
A/chicken/China/G2684/2014(H6N2)
A/duck/Vietnam/HU9-521/2018(H6N6)
A/waterfowl/Korea/S245/2016(H6N2)
A/duck/Vietnam/HU1-637/2014(H6N6)
A/duck/Viet Nam/QN-0518/2013(H6N6)
A/duck/Shantou/7568/2005(H6N2)
A/Eurasian Wigeon/Jiangxi/2018WB0158/2018 (H6N2)
A/Eurasian Wigeon/Jiangxi/2018WB0266/2018 (H6N2)
A/duck/Jiangxi/262/2013(mixed)
A/swine/Yangzhou/080/2009(H6N6)
A/chicken/Hunan/989/2005(H6N2)
A/duck/Fujian/629/2005(H6N2)
A/swine/Guangdong/K6/2010(H6N6)
A/wild duck/Shantou/2853/2003(H6N2)
A/duck/Shantou/339/2000(H6N2)
A/duck/Hong Kong/3174/99 (H6N2)
A/mallard duck/ALB/1151/1979(H6N9)
A/mallard/Maryland/881/2002(H6N2)
A/mallard/Wisconsin/857/1982(H6N8)
A/green-winged teal/Wisconsin/16/1974(H6N2)
A/duck/Minnesota/2/1975(H6N1)
A/Pacific black duck/Western Australia/2708/1979(H6N1)
A/mallard/Wisconsin/773/1982(H6N4)
A/turkey/Canada/1963(H6N8)
1925
1950
1975
2000
2025
